# Formal models for the study of the relationship between fluctuating asymmetry and fitness in humans

**DOI:** 10.1002/ajpa.24588

**Published:** 2022-07-21

**Authors:** Arodi Farrera

**Affiliations:** ^1^ Mathematical Modeling of Social Systems Department Institute for Research on Applied Mathematics and Systems, National Autonomous University of Mexico Mexico City Mexico

**Keywords:** causal inference, directed acyclic graphs, face, fitness, fluctuating asymmetry

## Abstract

**Objectives:**

To evaluate three of the main verbal models that have been proposed to explain the relationship between fluctuating asymmetry and fitness in humans: the “good genes,” the “good development,” and the “growth” hypotheses.

**Materials and Methods:**

A formal model was generated for each verbal model following three steps. First, based on the literature, a theoretical causal model and the theoretical object of inquiry were outlined. Second, an empirical causal model and the targets of inference were defined using observational data of facial asymmetries and life‐history traits related to fitness. Third, generalized linear models and causal inference were used as the estimation strategy.

**Results:**

The results suggest that the theoretical and empirical assumptions of the “good genes” hypothesis should be reformulated. The results were compatible with most of the empirical assumptions of “the good development” hypothesis but suggest that further discussion of its theoretical assumptions is needed. The results were less informative about the “growth” hypothesis, both theoretically and empirically. There was a positive association between facial fluctuating asymmetry and the number of offspring that was not compatible with any of the empirical causal models evaluated.

**Conclusions:**

Although the three hypotheses focus on different aspects of the link between asymmetry and fitness, their overlap opens the possibility of a unified theory on the subject. The results of this study make explicit which assumptions need to be updated and discussed, facilitating the advancement of this area of research. Overall, this study elucidates the potential benefit of using formal models for theory revision and development.

## 1 INTRODUCTION

Developmental stability (DS) is the ability of an organism to produce a consistent phenotype despite the environmental and genetic disturbances faced during development (Debat & David, 2001). This ability and its consequences are considered to have a fundamental role in the survival and/or reproduction of the individual and, therefore, to be an important part of fitness (Clarke, 1995). For decades, one of the most common measurements of DS has been the intra‐individual variability of paired bilateral traits, also known as fluctuating asymmetry (FA; Graham, 2021a; Palmer & Strobeck, 1992). The main argument for using FA is that since the sides of symmetrical organisms develop under identical genotypes and environments, the differences between them are mainly due to developmental noise (DN) or stochastic variation occurring during development (Hallgrímsson et al., 2002). This definition posits FA as an indicator of developmental precision and as a useful, cheap proxy of other more direct fitness estimates (e.g., Clarke, 1995). However, even though several hypotheses have been developed to explain these links and much research has been done on this topic, most of the evidence is inconclusive.

Particularly in humans, the evidence on the relationship between asymmetry and the components of the individual's health and fitness is ambiguous, as some studies have found an association (meta‐analysis: Møller & Thornhill, 1998; oxidative stress: Gangestad et al., 2010; attractiveness: Brown et al., 2008; sexual behavior: Kordsmeyer & Penke, 2017), while others have found little relationship between them (meta‐analysis: Palmer, 2000; van Dongen & Gangestad, 2011; attractiveness: Jones & Jaeger, 2019) or no relationship (attractiveness: Kleisner et al., 2017; health: Foo et al., 2017). Although most of this conflicting evidence can be attributed to the fact that FA is a weak measure of DS, methodological flaws or selective reporting and publication of mostly statistically significant results (e.g., Graham & Özener, 2016; Palmer, 1999; van Dongen & Gangestad, 2011), I argue that the lack of formal models for the hypotheses that explain these phenomena could also be a contributing factor to this problem.

As with other complex phenomena (Smaldino, 2020), the relationship between FA and fitness has been usually explained in descriptive terms. Although descriptive explanations (i.e., verbal models) are useful for delimiting the topic of interest or triggering the development of new ideas, we now know that the ambiguous way in which they are expressed makes it difficult to, for example, clearly establish how hypotheses relate to observed data or to recognize whether a result constitutes evidence for or against a given hypothesis (Smaldino, 2017). Therefore, it is possible that the hypotheses that have been proposed thus far have been ill‐defined (e.g., by confusing hypothesis with its predictions, Strode, 2015) or expressed in such an ambiguous way that they are obscuring the already weak evidence on FA as an indicator of developmental precision, thus hindering the interpretation of the link between FA and any health and fitness outcome. Furthermore, ambiguous explanations make it difficult to update hypotheses, as it is unclear how new ideas and assumptions connect to old ones, or how to use new results for theory revision and development. In this way, continuing to test outdated hypotheses could also be a contributing factor to the ambiguous evidence found in the study of FA and fitness.

Formal models address some of the problems related to descriptive explanations by specifying in precise terms which variables are relevant to a given topic and our assumptions about how they are related (Robinaugh et al., 2021; Smaldino, 2020). In humans, three main verbal models have been proposed to explain the relationship between FA and fitness: the “good genes,” the “good development,” and the “growth” hypotheses; but to my knowledge, no formal model has been developed on this relationship, nor have all three hypotheses been evaluated and compared simultaneously. As a first step in this direction, the aim of this contribution is twofold: to propose formal models for these common hypotheses and test these formal models in the particular case of facial asymmetries and reproductive success.

### 1.1 A brief introduction to formal models

Both verbal and formal models articulate some aspect (e.g., components, relationship between components, consequences) of a complex phenomenon of interest (see Frigg & Hartmann, 2020), but while the former does so using descriptive explanations, the latter does so through graphical representations (e.g., Rohrer, 2018) and mathematical or computational modeling (Smaldino, 2020). By making the research question explicit, as well as the assumptions about what components are (or are not) relevant and how they connect to each other (see Smaldino, 2020; Robinaugh et al., 2021 for an introduction), formal models make it possible for research goals, methodology, and results to align. Although there is no unique procedure to generate these models, there are two approaches that help in this task: the estimand framework and the causal framework.

Traditionally, an estimand defines a target quantity to be estimated, while an estimator and an estimate refer, respectively, to the method used to obtain an approximation of this target and the specific value obtained when this method is applied to actual data (e.g., Little & Lewis, 2021). The estimand framework (see Lundberg et al., 2021 for an introduction) considers an additional distinction between theoretical and empirical estimands that improves the link between theory and evidence by clearly delineating the conceptual and empirical parts of the argument and accounting for cases where these estimands are not equivalent. This framework allows us to explicitly state what we try to know or describe (i.e., theoretical estimand: quantity of theoretical interest), what we can actually learn from available data and procedures (i.e., empirical estimand: quantity of practical interest), and how we can learn from data (estimation strategy).

Briefly, the theoretical estimand defines in precise terms the target of inquiry by formalizing the quantity most relevant to the theory and the target population over which to draw inferences. Since it is derived from theory, it can account for observable and unobservable variables (e.g., missing data). An empirical estimand, on the other hand, defines the quantity that can be recovered from observed data only, and thus informs us about the theoretical estimand under specific assumptions (e.g., convenience sample). The last component of this framework is the estimation strategy or the process that will be used to learn about the empirical estimand, which includes the estimator and estimate. Among other things, it is sought (see Wasserstein et al., 2019 for an introduction) that rather than estimation strategies that rely on making dichotomous inferences about the presence or absence of the effects of interest (e.g., using null hypothesis significance testing statistical or Bayes factor) and on reporting and interpreting point estimates, estimation strategies focus on estimating the direction and size of these effects, and on embracing uncertainty, for example, by reporting frequentist confidence intervals or their Bayesian counterparts, credible intervals (Berner & Amrhein, 2022; Smith, 2018).

The causal framework (see Hernán & Robins, 2020; Pearl & Mackenzie, 2018, for an introduction) on the other hand, allows us to explicitly estate our assumptions about how the theoretical and empirical estimands connect to each other and to other variables, and it allows us to identify causal effects, rather than correlations, between these variables. One popular way of representing a causal structure is through directed acyclic graphs (DAGs). In these graphs, nodes represent variables and causal effects are represented by arrows pointing away from one variable to another (e.g., *X* → *Y*, meaning *X* affects *Y*). The difference between DAGs and other ways of encoding the causal relationship, such as structural equation models (SEMs), is that while the former encodes the qualitative relationship between them, the latter specifically encodes the form (e.g., linear, additive relationships) of said relationship (Rohrer, 2018).

At a practical level, the assumptions made in a DAG can be used in observational studies for causal inference, that is, to identify causal effects between variables, rather than correlations (see Rohrer, 2018 for an introduction). Assuming that the DAG captures the true causal structure, a set of rules can be applied to determine the sufficient set of variables needed to estimate the actual causal effect of *X* on *Y*. These rules eliminate problems commonly present in observational studies that can bias this estimate or induce spurious associations, such as confounding (e.g., Westreich & Greenland, 2013) or collider bias (e.g., Schneider, 2020).

One of the advantages of the combined approach of estimands and causal framework is that the research question is no longer bound by statistical procedures. That is, rather than being used as an equivalent to, for example, scientific inference, importance, or decision making (Hubbard et al., 2019; Navarro, 2019; Wasserstein et al., 2019), statistical inference plays a limited role in this scientific process (i.e., a component of the estimation strategy that intends to learn about the empirical estimand). Other advantages are that methodological choices and conclusions are framed transparently within the corresponding empirical estimand (as opposed to theoretical ones) and that it provides a basis for cumulative knowledge on the subject (see Lundberg et al., 2021).

In the remainder of this article, I will use this combined framework to study the relationship between asymmetry and fitness. First, based on the literature, I outline, for each verbal model proposed to explain the relationship between FA and fitness (i.e., the “good genes,” the “good development,” and the “growth” hypotheses), a theoretical causal model of this relationship and establish what the theoretical object of inquiry (i.e., theoretical estimand) is. Then, from these general models, I outline the empirical causal model and the target of inference (i.e., empirical estimand) that is the focus of the present study, and the estimation strategy used.

### 1.2 Theoretical assumptions: Fluctuating asymmetry and fitness in humans

The definition of FA entails two different interpretations of what an increase in FA means (Klingenberg, 2019; van Dongen & Gangestad, 2011). The first one considers that higher FA values result from the inability of an organism to buffer its development against stochastic variation. In contrast, the second considers that higher values are the result of prolonged or frequent exposure to non‐genetic perturbations. For example, an individual may express greater FA because these perturbations occurred during a sensitive window of development (e.g., Oxilia et al., 2021), or because the symmetric structure was continuously exposed to asymmetric environments (chewing: Martinez‐Gomis et al., 2009; motor tasks: Aune et al., 2016).

**FIGURE 1:**
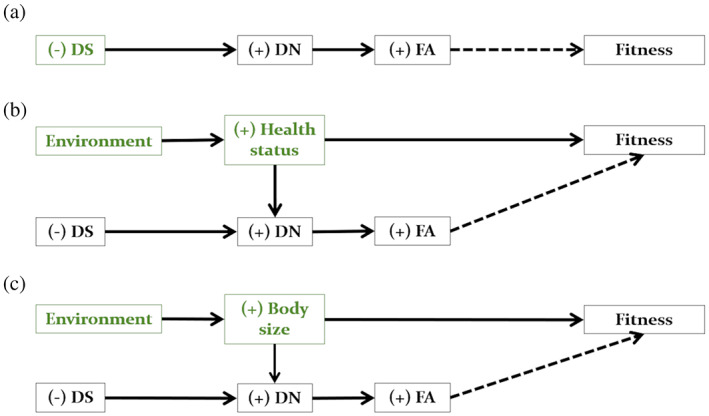
Theoretical causal models of the hypotheses on the relationship between fluctuating asymmetry and fitness. Arrows represent a causal effect from one variable (start) to another (end). The dashed arrows depict the theoretical object of inquiry: The relationship between FA and fitness. Following the literature, each model represents the corresponding set of causal assumptions. (a) *Good genes hypothesis*: Reduced DS leads to increased DN and asymmetry. FA is an indicator of the genetic quality (green) of an individual. (b) *Good development hypothesis*: Reduced DS leads to increased DN, but so does a poor health status. Both lead to increased asymmetry. The effect of FA on fitness is confounded (sensu causal inference: FA ← DN ← health status → fitness; Rohrer, 2018) by the effect of the internal and external environment in which the individual develops (green). (c) *Growth hypothesis*: Both reduced DS and larger size lead to an increase in DN. The effect of FA on fitness is also confounded (FA ← DN ← body size → fitness) by the effect of the internal and external environment (green) in which the individual develops. DS, developmental stability; DN, developmental noise; FA, fluctuating asymmetry. See main text for more details.

Both interpretations convey different assumptions. Specifically, while the first one focuses on the individual's attributes, the other highlights the environmental context. These different perspectives in turn result in three different descriptive explanations of the mechanisms that explain the relationship between FA and fitness. Figure 1 shows the theoretical causal model derived from each of these verbal models.

Based on the first explanation, the “good genes” hypothesis (Figure 1a) proposes that FA is a signal of good genes or genetic quality, that is, a biomarker with which individuals assess the efficiency of potential mates' buffering mechanisms (Jones et al., 2001; Thornhill & Gangestad, 1993). Because perfect symmetry imposes high physiological costs, it can only be achieved by individuals in excellent condition (Thornhill & Gangestad, 1993), especially if it is expressed in sexual traits that already convey a cost compared to nonsexual traits (Møller, 1991). According to this hypothesis, FA should be related to fitness components (e.g., attractiveness or mating success) because the selection of mates with symmetrical traits would enhance offspring viability (e.g., Møller et al., 1995; Thornhill & Gangestad, 1994).

The “good development” hypothesis (Figure 1b) relies on the second explanation to propose that FA is a biomarker of developmental plasticity under conditions of energetic stress. Specifically, from a life history perspective, the extra energy requirements associated with, for example, compensatory growth (Wells et al., 2006) or metabolically expensive tissues (Longman et al., 2021) would exert a cost in the development of symmetrical traits. In this case, FA would be indirectly related to fitness through the individual's health status because, similar to the previous hypothesis, only individuals in good conditions could afford a symmetric phenotype in harsh circumstances.

A third explanation (Figure 1c) is one in which the expression of FA during development does not require additional costs because it is tightly associated with the phenotype of the individual. Specifically, since traits that grow for longer periods and that are larger will have more opportunities for asymmetry (Leung, 1998; Palmer & Stobeck, 2003), FA in these phenotypes will be related to body size and will be amplified by environmental perturbations affecting growth. In these traits, the relationship between FA and fitness will be confounded if body size also influences the latter (e.g., Walker & Hamilton, 2008). Then, according to this “growth” hypothesis, size variation and FA may independently reflect meaningful information about the development of the individual and in those phenotypes in which both traits covary, FA and its consequences on fitness become a combination of the effects of DS, allometry, and environment (e.g., Palmer & Stobeck, 2003). In other words, this hypothesis assumes that FA is indirectly related to fitness through body size but does not exclude the possibility that FA directly affects fitness.

The variety of explanations represented by these hypotheses reflects the implicit complexity that exists in this topic; however, the theoretical estimand remains the same: the causal effect of FA on fitness. In the following, I test these hypotheses focusing on the widely studied topic of facial asymmetries.

## 2 MATERIALS AND METHODS

To facilitate the comparison of the three hypotheses, I use observational data from a multigenerational pedigree sample that includes facial FA values and life‐history traits related to fitness. Below, for each hypothesis, I detail the study population and the variables of interest and outline the relevant empirical estimands.

### 2.1 Sample description

The sample included 314 subjects (207 females, 107 males; mean age = 38.47, *SD* = 18.02), distributed in 78 extended and nuclear families, with an average of three members per family (range = 0–11). This dataset was collected in Chiapas province in Mexico. Volunteers who lived in the same geographic area throughout their lives, without previous facial surgery, craniofacial trauma, congenital anomalies, or orthodontic treatment were included in this study. Informed consent was signed by each participant before personal and phenotypic data were collected (Farrera, 2014).

### 2.2 Data collection

#### 2.2.1 3D photogrammetry imaging technique

The 3D facial shape was captured using photogrammetric methods applied to a series consisting of five separate digital photographs from different angles (left side, left angle: 45°, frontal, right angle: 135°, and right side). All photos were taken at a constant distance of 1.5 m with a standardized photographic protocol described in detail previously (see Quinto‐Sánchez et al., 2015). Special care was taken with the hair and earrings. The 3D coordinates were obtained using the software Photomodeler (https://www.photomodeler.com/; Eos Systems, Vancouver, Canada), following the standard recommendations for quality and accuracy of the software. The distance chelion‐chelion, measured directly on the individuals using a standard anthropometric caliper, was used as a scale factor.

**FIGURE 2:**
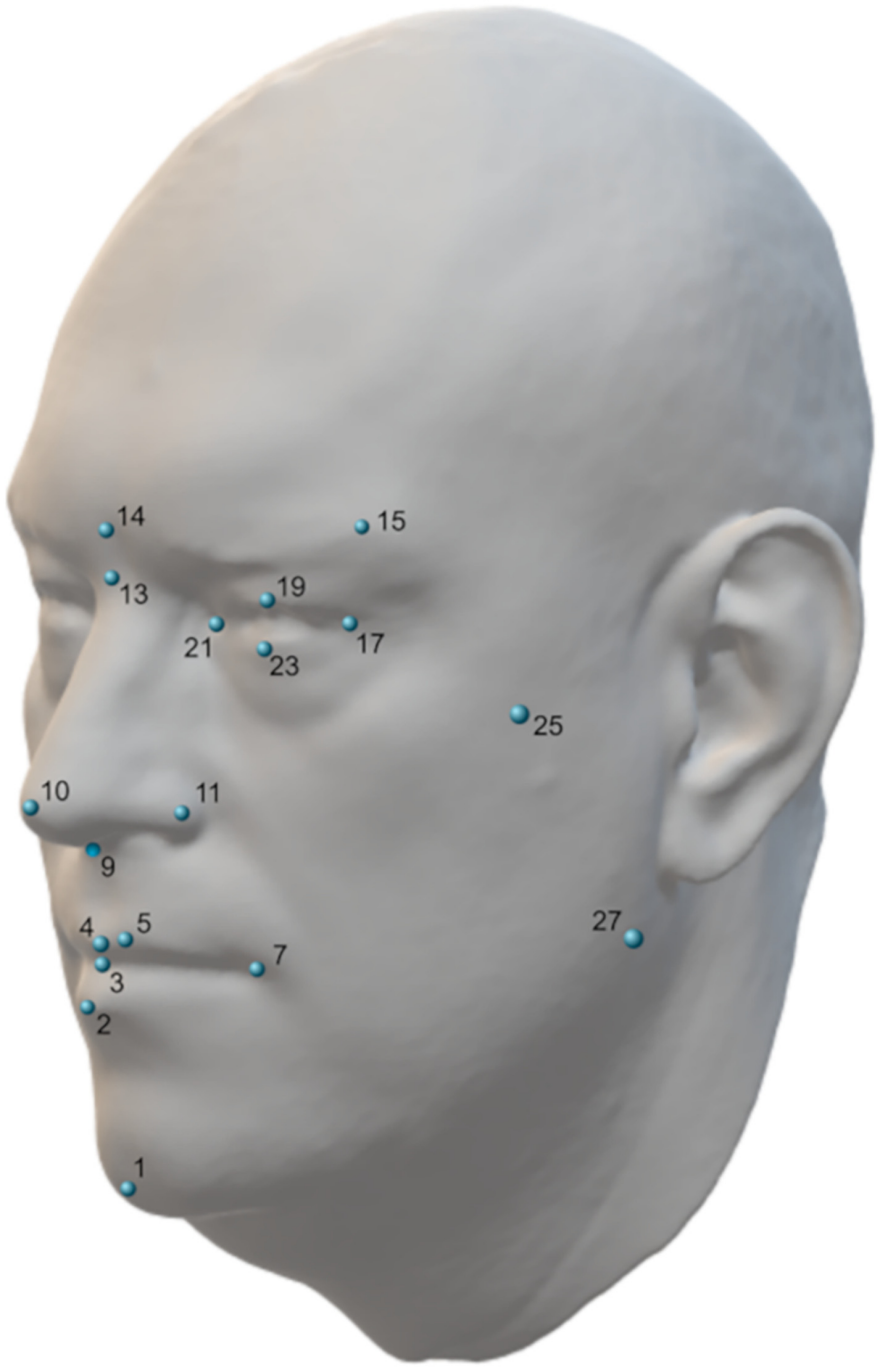
3D facial scan with digitized landmarks. (1) Gnathion, (2) Labrale inferius, (3) Stomion, (4) Labrale superius, (5, 6) crista philtri, (7, 8) Cheilion, (9) Subnasale, (10) Pronasale, (11, 12) Alare, (13) Nasion, (14) Glabella, (15, 16) Frontotemporale, (17, 18) Exocanthion, (19, 20) Palpebra superior, (21, 22) Endocanthion, (23, 24) Palpebra inferior, (25, 26) Zygion, (27, 28) Gonion. (Artec3D, 2021).

Forty‐two landmarks (17 bilateral and 8 sagittal landmarks) were placed on the photographs trying to avoid data redundancy and following standard terminology (see Figure 2). The points that needed to be located by palpation were marked in situ with a sticker (zygion, gonion, gnathion, frontotemporale). A subsample of 61 individuals was digitized a second time to evaluate intraobserver variation. A Procrustes ANOVA analysis was performed in this subsample to assess the repeatability of data acquisition in different sessions (Klingenberg & McIntyre, 1998). The results of this Procrustes ANOVA (Table 1) show that the measurement error is one order of magnitude smaller than fluctuating asymmetry. Given the overall goal of the manuscript, this measurement error was considered acceptable for subsequent analysis.

**TABLE 1:** Results of Procrustes ANOVA displaying the measurement error in shape variables

Effect	SS	MS	*df*	*F*	*p* (Param.)
Individual	0.50767632	0.0001239747	4095	6.20	<0.0001
Directional asymmetry	0.00863661	0.0001542253	56	7.72	<0.0001
Fluctuating asymmetry	0.07273774	0.0000199829	3640	5.12	<0.0001
Measurement error	0.03065837	0.0000039035	7854		

#### 2.2.2 Measurements

##### Fluctuating asymmetry

The 3D coordinates of all landmarks were superimposed using the generalized Procrustes analysis (GPA) in the MorphoJ software (Klingenberg, 2011). This procedure standardizes the configurations of landmarks by eliminating differences in position, size, and orientation. For landmark configurations with object symmetry like the face, the Procrustes fit is performed on the original configurations and their mirror images. The individual scores of FA in units of Mahalanobis distance are obtained from this procedure as the variation of individual asymmetries around the mean asymmetries. These individual scores indicate the magnitude of FA (i.e., the higher the score, the higher the FA) independently of directional asymmetry (Klingenberg, 2015). These scores (mean = 7.49, *SD* = 0.91; range = 5.40–10.14) were used in subsequent analyses.

##### Life history traits

Data on life history traits were only available on a subsample of female individuals (*n* = 207; mean age = 39.6, *SD* = 17.4). The number of offspring (mean = 2.71, *SD* = 2.41; range = 0–11) was considered as a measure of fitness, and the adult height (mean = 153.2, *SD* = 5.90; range = 139–167) of the individual as a proxy of their health status (see empirical causal model below).

### 2.3 Empirical assumptions: Facial fluctuating asymmetry and the number of offspring

**H1.**  The “good genes” hypothesis: FA encoding genetic quality.

**FIGURE 3:**
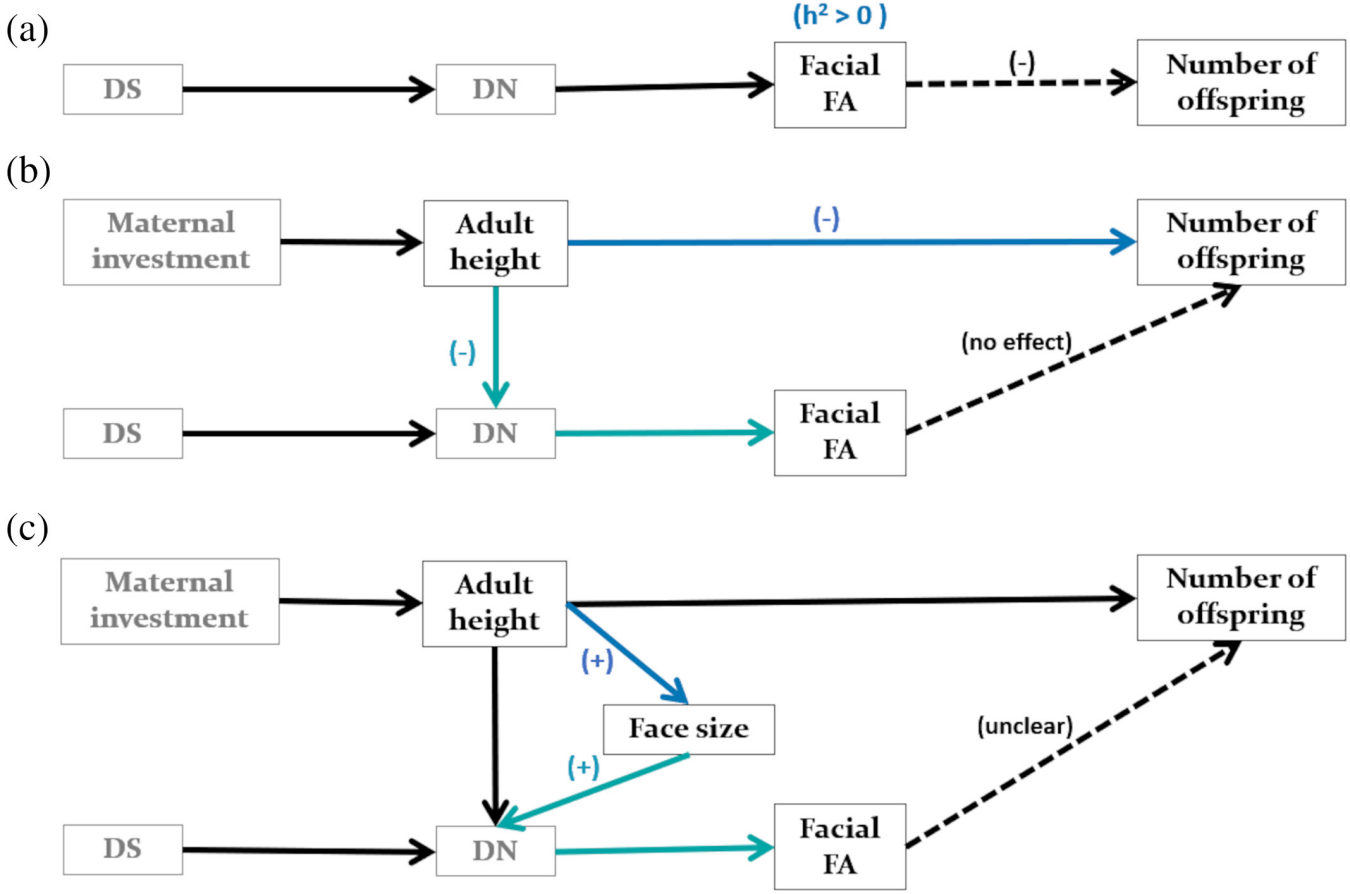
Empirical causal models of the relationship between facial fluctuating asymmetry and reproductive success (i.e., number of offspring) according to the set of assumptions derived from the (a) “good genes,” (b) “good development” and (c) “growth” hypotheses. Arrows represent a causal effect from one variable (start) to another (end). Dashed arrows depict the main empirical estimand: The causal effect of facial FA on the number of offspring. Secondary empirical estimands are shown in blue. Predictions for each empirical estimand are shown in parentheses. Variables in black correspond to the dataset analyzed, while those in gray represent unmeasured variables. DS, developmental stability; DN, developmental noise; FA, fluctuating asymmetry. See main text for more details.

This hypothesis assumes that since facial asymmetries measure DS, FA can be used as a biomarker of genetic quality. Furthermore, it assumes that such an association will impact the reproductive success of the individual, through attractiveness (Møller et al., 1995). The empirical causal model shown in Figure 3a outlines these assumptions. Accordingly, the main empirical estimand is the direct causal effect of facial FA on reproductive success (i.e., number of offspring). This empirical causal model predicts a negative relationship between these variables (see Møller & Thornhill, 1998): individuals with higher facial FA values (i.e., less attractive) would have fewer offspring.

Additionally, this hypothesis assumes that DS, and therefore, FA, must be partly under genetic control for it to respond to selection (i.e., mate choice; Leamy & Klingenberg, 2005). The idea is that, if FA has genetic variability, closely related individuals will have FA values more similar than those who are not. Therefore, a secondary empirical estimand associated with this hypothesis is the heritability (*h* ^2^) of FA, expecting values greater than 0 (Figure 3a). I estimated the heritability of FA using a generalized linear mixed model via Markov chain Monte Carlo methods using the MCMCglmm, R package (Hadfield, 2010). The additive genetic variance and the residual environmental variance of FA were estimated using the pedigree information of the total sample. Weak priors were set for both variance components and, based on the empirical causal model (Figure 3a) of this hypothesis, no fixed effects were included. The MCMCglmm was run for 10,000,000 iterations with a burn‐in period of 2000 iterations and a thinning interval of 1000.

**H2.**  The “good development” hypothesis: FA encoding developmental plasticity.

The empirical causal model for this hypothesis was generated based on the work of Wells (2018, 2019), which incorporates the maternal phenotype as the main environmental factor that impacts the development of the offspring. According to this verbal model greater maternal investment in both pregnancy and lactation would favor in the offspring the allocation of energy to somatic growth and maintenance and, therefore, would result in large adult size. In this scenario, offspring quality is favored over quantity. In contrast, a reduced maternal investment would favor the allocation of energy to reproduction, which would result in small adult size. If the post‐natal energy supply improves in the latter scenario, the small size will also be accompanied by elevated fat stores and an increased risk of disease. In both scenarios, offspring quantity would be favored over quality.

Based on these intergenerational maternal effects, Figure 3b shows the empirical causal model generated for the “good development” hypothesis. In contrast to the previous hypothesis, the model does not predict a direct causal effect, instead, the main empirical estimand in this model is the indirect causal effect of facial FA on the number of offspring through adult size. A secondary empirical estimand derived from this model is the causal effect of adult height on the reproductive success of the individual (e.g., Wells, 2018). Therefore, a negative relationship between variables is expected: individuals who developed in poor conditions (i.e., short stature) would have more offspring and vice versa. Another secondary empirical estimand derived is the causal effect of adult height on facial FA through developmental noise. Because individuals who developed in suboptimal conditions (i.e., short stature) could not afford symmetric traits (e.g., Longman et al., 2021), a negative relationship between these variables is expected: shorter individuals would have greater facial FA values (e.g., Özener & Ertuğrul, 2011).

**H3.**  The “growth” hypothesis: FA as a by‐product of body size.

This hypothesis is tested using the previous empirical causal model with two additional assumptions (Figure 3c): that body height and face size are positively allometrically related in adults (e.g., Mitteroecker et al., 2013) and that face size has a causal effect on FA because more growth gives more opportunities for asymmetry (e.g., Palmer & Stobeck, 2003). The literature is not clear on whether the effect of facial FA on the number of offspring is direct or indirect, so this model has two main empirical estimands: a direct causal effect as in the first model or an indirect causal effect as in the second one. The first assumption predicts an association between these variables, while the second predicts no direct causal effect. There are also two secondary empirical estimands: the causal effect of height on face size, and the effect of face size on facial FA. In both cases, a positive relationship is expected: taller individuals will exhibit bigger faces and individuals with larger faces will exhibit larger facial FA values, respectively.

### 2.4 Estimation strategy

The main empirical estimand is the same across hypotheses: the causal effect of facial FA on the number of offspring, conditional on the empirical causal structure assumed (empirical DAG). Given that the estimation of this effect involves life‐history traits, all three hypotheses were evaluated using the subsample of females.

The strategy to estimate the causal effect of facial FA on the number of offspring given the corresponding empirical causal model was, first, to use an automated causal modeling tool (R package: *Dagitty*, Textor et al., 2016) to identify the right covariates. This tool allows one to test whether the assumptions encoded in the DAGs are consistent with the data and to test whether the inferences made in the original DAGs are valid for a range of different but statistically similar DAGs (Textor et al., 2016). The DAG that resulted from this process was consistent with the data and robust against different causal specifications, and therefore, used in the following analysis. Then this DAG was used to identify, for each empirical estimand, the set of covariates to be adjusted for in the statistical models. Poisson regression was used for the analysis of count data, whereas linear regression was used for continuous data.

For H1, conditional on the empirical DAG, the Poisson regression model estimated the effect of facial FA on the number of offspring without adjusting for any variable.

For H2, conditional on the empirical DAG, the causal effect of facial FA on the number of offspring was estimated using a Poisson regression model adjusted for height. On the other hand, the effect of height on the number of offspring was estimated using a Poisson regression but given the DAG, no adjustment was necessary. To estimate the effect of height on facial FA assumed in this hypothesis, a linear model was fit without adjusting for any covariate.

For H3, conditional on the empirical DAG, the Poisson regression model between facial FA and the number of offspring was fit both without adjustment and adjusting for height. Additionally, the effect of height on facial size was estimated using a linear model without adjusting for any covariate. Finally, the effect of face size on facial FA was estimated using a linear model adjusted for height.

Continuous variables were standardized before the analysis. Data analysis was conducted using the R language and environment (R Core Team, 2020), version 1.2.5033 via the RStudio integrated development environment.

## 3 RESULTS

The results for H1 show that there was a positive effect (*β* = 0.323) of facial FA on the number of offspring, with possible values for this parameter that were most compatible with the data ranging from 0.244 to 0.402 (95% confidence interval). In other words, conditional on the DAG, individuals with higher asymmetries have more children. Additionally, the generalized linear mixed model provided a mean heritability of facial FA close to zero (*h* ^2^ = 0.046) with possible values ranging from 0.0002 to 0.174 (95% confidence interval), which means that almost none or little of the phenotypic variation in FA is explained by genetic variation.

The results for H2 show that there was a positive effect (*β* = 0.294) of facial FA on the number of offspring conditional on the individual's adult height, with possible values for this parameter ranging from 0.213 to 0.374 (95% confidence interval). This means that individuals with higher FA values for their height have more offspring. In addition, there was a negative effect (*β* = −0.275) of the adult height on the number of offspring, with possible values ranging from −0.359 to −0.191 (95% confidence interval). In other words, those individuals who are taller have fewer offspring. Finally, there was a negative effect (*β* = −0.13) of height on facial FA, with possible values ranging from −0.266 to 0.006 (95% confidence interval). This means that taller individuals exhibit lower facial FA.

The results for H3 show that the direct and indirect effects of facial FA on the number of offspring calculated under this model are the same as those effects obtained in the first and second hypotheses, respectively. Moreover, there was a very low positive effect of height on face size (*β* = 0.01) with possible values for this parameter that were most compatible with the data in a relatively wide range from −0.127 to 0.148 (95% confidence interval). Finally, the results show a very low negative effect (*β* = −0.04) of face size on facial FA with possible values in a relatively wide range from −0.179 to 0.093 (95% confidence interval).

## 4 DISCUSSION

In this contribution, I evaluated three of the most common verbal models used to understand the relationship between FA and fitness in humans: the “good genes,” the “good development,” and the “growth” hypotheses. For this purpose, I generated formal models (i.e., estimands and causal frameworks) for each hypothesis and tested them in the particular case of facial asymmetries and reproductive success.

### 4.1 Theoretical assumptions

The present study shows that even if the approaches are different, some of the theoretical assumptions overlap across hypotheses (Figure 1), opening the opportunity for a unified formal model. Nonetheless, they show differences in two key assumptions. First, these hypotheses differ in whether they consider that FA reflects some cost to the individual, distinguishing between FA as a reliable signal of DS that reflects the quality of the individual (H1 and H2: symmetrical traits are costly) and as a reliable signal that requires no additional cost because it is tightly associated with some attribute of the individual (H3: allometric constraints that link body size and FA). This distinction has been discussed mainly in the framework of signaling theory (Barker et al., 2019), but in the context of human asymmetries and fitness, this discussion is currently problematic primarily because the way these concepts have been applied overlooks recent conceptual advances.

From the framework of signaling theory, attributes other than physiological information are recognized as signals (e.g., embodied capital or noetic attributes, Barker et al., 2019). A broader concept like this would allow for more comprehensive verbal models of the relationship between FA and fitness in humans, in which cultural practices such as the use of makeup (Killian et al., 2018), and social norms like standards of beauty (Kleisner et al., 2017) are also included in the interpretation and scope of the research. Signaling theory also recognizes that the way multiple signals are integrated with each other and with socioecological factors is an important source of information (Patricelli & Hebets, 2016). This would promote studying asymmetry along with other types of signals, as has been done during the last decade on topics such as mate choice (Jones & Jaeger, 2019; van Dogen et al., 2020) or the individual's health status (Foo et al., 2017; Mogilski & Welling, 2017). Addressing multiple signals as an integrated signaling phenotype or explaining how they are theoretically related to each other (e.g., Luoto et al., 2021) could improve and extend our understanding of the topic. Moreover, instead of being considered a static measurement (i.e., values computed at one point in time), individual asymmetries could be explored over different timescales. In the dynamic context of face‐to‐face interaction, for instance, asymmetric facial movement can be perceived as unattractive, regardless of the static asymmetry score of the individual (Hughes & Aung, 2018) because, for example, it conveys information about the sender's age (Kamachi et al., 2019). Taking into account that the causes and effects of asymmetry can be different in static and dynamic contexts could also clarify some of the contradictory evidence on the subject.

Another theoretical assumption in which the hypotheses evaluated differ is whether they highlight the role of developmental plasticity (i.e., phenotypic adjustments in response to the environment) on the expression of phenotypic variation and, particularly, on the production of asymmetric traits. Specifically, this assumption differentiates between research on FA variation that focuses on its genetic basis (H1: symmetry reflects good genes) and research that focuses on the development pathways that lead to such within‐individual variation (H2 and H3: symmetry reflects the interplay between the organism and its circumstances). Although the former ignores the idea that has been present since the 1980s in the field of evolutionary developmental biology (Müller, 2007) that the influence of genotype on the phenotype is structured by developmental processes, the role of development in the latter is not entirely clear either. New verbal and formal models with a different set of theoretical assumptions are needed to get a better, refined representation of the role of development in the relationship between FA and fitness in humans.

### 4.2 Empirical assumptions

This study also shows some similarities and differences between hypotheses when the results are compared with the expectations derived from the empirical assumptions. In the case of the “good genes” hypothesis, the corresponding empirical causal model expects that individuals with greater facial FA values have less reproductive success than individuals with less asymmetry. Moreover, it expects that part of the phenotypic variance of facial FA is explained by genetic variation. In contrast, I found that individuals with greater facial FA values have more offspring and a heritability close to zero (i.e., almost none or little facial FA variation is explained by genetic variation). The latter result is the first report of heritability of FA in the human face and is consistent with previous research showing very low or no heritability of other traits in humans and other species (Johnson et al., 2008; Leamy & Klingenberg, 2005). These results suggest that the empirical causal model for this hypothesis needs to be revised and refined.

The empirical causal model derived from the “good development” hypothesis posits that because FA is the result of poor health, no direct link should be found between facial FA and the number of offspring. In contrast, the results showed a positive effect of facial FA on the number of offspring. On the other hand, based on the intergenerational maternal effect (Wells, 2018), this empirical causal model assumes that the short stature of some individuals is the result of a suboptimal maternal niche and that individuals who develop under these conditions may favor quantity over quality of offspring, and vice versa. The results of this study were compatible with this assumption. Specifically, it was found that, regardless of asymmetry, individuals with poor health status (measured as adult height) had more children, an effect reported in some previous studies (e.g., Krzyzanowska et al., 2015), but not in others (e.g., Helle, 2008). The results were also compatible with the expected negative effect of height on facial FA in this hypothesis, an effect reported in previous studies (Kirchengast, 2019; Özener & Ertuğrul, 2011) using FA measurements of non‐facial traits. In other words, these results are compatible with most of the assumptions derived from the empirical causal model for this hypothesis, except for the assumption of no direct link between facial FA and the number of offspring, which should be refined to include potential mechanisms that may explain the relationship between these variables.

The empirical assumptions of the “growth” hypothesis are unclear as to whether facial FA directly and/or indirectly influences the number of offspring, what would be the expected direction of this effect, or what mechanism would be responsible. Therefore, it is currently not possible to interpret the results obtained on this assumption. Nonetheless, this empirical causal model posits two additional assumptions. First, that body and face size are allometrically related in adults and that facial FA is a by‐product of individual growth. In contrast to previous studies (e.g., Gateño et al., 2018; Mitteroecker et al., 2013), the results were compatible, with high uncertainty, with an effect close to zero. These results suggest that more discussion is needed on the empirical causal model derived for this hypothesis.

In all hypotheses, I found a positive association between facial FA and the number of offspring, which is not consistent with any of the three empirical causal models evaluated. This result suggests that additional explanatory variables should be formally included in these models to further understand and test this relationship. One candidate variable could be the age‐dependent pattern of FA expression (e.g., Wilson & Manning, 1996). Since facial FA can be a by‐product of soft tissue aging, older individuals may express higher values. Further, this link could be related to the number of offspring in two ways. First, in line with the “good development” hypothesis, since reproduction takes time and considerable metabolic demands, individuals who have reproduced more and are older may also be more asymmetric. Second, in line with the “growth” hypothesis, fully developed (older and bigger) and therefore more asymmetric individuals could be those who have also had more opportunities to reproduce. Datasets collected specifically for testing these verbal models and updated formal models are needed to confirm the role of aging or any other variable outside those proposed in this work.

There are at least two factors related to the estimation strategy that limit the interpretation of these results (section 4.2). One of them is the sample over which inferences were drawn. The dataset used in this study was not explicitly collected to answer the theoretical object of inquiry (i.e., the relationship between FA and fitness), and thus, the empirical causal models were designed after data collection, instead of before as required to warrant causal claims (Rohrer, 2018). Other potential factors are related to bias in the computation of FA values, which have been extensively reviewed elsewhere (Graham, 2021b; Graham et al., 2010), including the presence of other forms of asymmetry, measurement error, or mixtures of additive and multiplicative errors. These limitations suggest that these results (section 4.1) must be replicated using more rigorous estimation strategies and other databases that allow comparing the three hypotheses.

Future studies could further benefit from revising, in light of theory development, the statistical practice associated with FA. For instance, rethinking isolated FA values as a target of inquiry when evidence suggests that in some contexts it is common to find different forms of asymmetry together (e.g., human face: Farrera et al., 2015; Quinto‐Sánchez et al., 2015). Formal models of descriptive explanations that instead address the dynamics that could give rise to patterns of asymmetric mixtures (e.g., Graham et al., 1993; Hallgrímsson, 1998) could shed new light on the topic or clarify existent evidence.

## 5 CONCLUSIONS

The overlap between theoretical and empirical assumptions across hypotheses supports the idea that the relationship between asymmetric variation and fitness cannot be understood using only one of them, but rather requires a general model that integrates different explanations on this topic. However, before a unified framework can be developed, several theoretical and empirical assumptions of the three most common hypotheses on this subject need to be revised and updated.

Altogether, the results of this study suggest that the “good genes” hypothesis needs to be reformulated for several reasons. The first of which is because its theoretical assumptions have not been revised and updated since its development in the 1990s, despite conceptual advances in signaling theory and evolutionary development to understand phenotypic variation. Another reason is that, although early studies showed support for this hypothesis (e.g., Møller & Thornhill, 1998; Perret et al., 1999), the present study and accumulated evidence show little or no support (e.g., Foo et al., 2017; Kleisner et al., 2017; Kočnara et al., 2019; Kruuk et al., 2003; Palmer, 1999; Zheng et al., 2021), particularly under naturalistic settings (Jones & Jaeger, 2019).

The results of this study were compatible with most of the empirical assumptions made for the “good development” hypothesis. In contrast, it is not yet clear whether its theoretical assumptions hold, and to what extent they need to be extended or refined, mainly because despite being formulated in the early 2000s (Wells et al., 2006), only a few studies to date have addressed it directly (Kirchengast, 2019; Longman et al., 2021; Özener & Ertuğrul, 2011). Finally, the present study is less informative about the “growth” hypothesis because the literature is not clear about the theoretical assumptions on the relationship between FA and fitness. This makes it impossible to further assess the empirical assumptions derived from them. Additionally, the results of this study were not compatible with the secondary empirical assumptions derived from this hypothesis.

## AUTHOR CONTRIBUTIONS

**Arodi Farrera:** Conceptualization (lead); data curation (lead); formal analysis (lead); investigation (lead); methodology (lead); writing – original draft (lead).

## FUNDING INFORMATION

The author was supported by the Postdoctoral Fellowship program DGAPA‐UNAM.

## CONFLICT OF INTEREST

The author declares no conflict of interest.

## Data Availability

The source code for generating all the analyses in section 2.4 is available here: https://github.com/arodifr/FA_fitness.git. Due to the nature of the pedigree data, the data used for section 2.3. H1 are not available.
